# Imaging Characteristics of Primary Mucinous Cystadenocarcinoma of the Breast: A Case Report and Literature Review

**DOI:** 10.2174/0115734056345716241213075234

**Published:** 2025-01-02

**Authors:** Yizhong Bian, Lei Xu, Yibo Zhou, Jizhen Li

**Affiliations:** 1 Department of Ultrasound Medicine, Affiliated Jinhua Hospital Zhejiang University School of Medicine, Jinhua, Zhejiang, 32100, P. R. China; 2 Department of Pathology, Affiliated Jinhua Hospital Zhejiang University School of Medicine, Jinhua, Zhejiang, 32100, P. R. China

**Keywords:** Primary mucinous cystadenocarcinoma of the breast, Imaging diagnosis, Case report, Examination, Literature review, Time-signal intensity curve (TIC)

## Abstract

**Introduction:**

Mucinous Cystadenocarcinoma (MCA) of the breast remains a relatively rare condition, and to date, there is no systematic summary of its imaging manifestations. Therefore, this report presents a detailed account of the diagnosis and treatment of mucinous cystadenocarcinoma in a 40-year-old woman, with a particular focus on imaging findings. Additionally, we conducted a comprehensive literature review on this disease and summarized its key imaging features. This manuscript provides valuable insights and methodologies for the accurate diagnosis of mucinous cystadenocarcinoma.

**Case Presentation:**

We report a 40-year-old premenopausal woman who discovered multiple cysts in her left breast five years ago. Over the past two years, the size of these tumors has increased. Ultrasound examination indicated that the cysts had grown to 27 x 17mm. Following a puncture, the cysts were confirmed to be benign and were not monitored regularly. A year later, the patient's mass in the left breast increased, and an ultrasound exam indicated a suspicious mixed echo area in the upper outer quadrant, suggestive of a malignant lesion. Mammography showed amorphous suspicious calcifications in the lesion area, distributed in segments. Contrast-enhanced magnetic resonance imaging displayed non-mass-type enhancement of the lesion, with a dynamic enhanced imaging time-signal intensity curve (TIC) showing a rapidly rising plateau pattern. Postoperative pathology confirmed invasive carcinoma of the left breast along with mucinous cystadenocarcinoma. Four months after surgery, the patient developed multiple abnormal lymph nodes in the left axilla, which were confirmed to be metastasis upon pathology examination. Following radiotherapy, the patient's condition remained stable during the follow-up period.

**Conclusion:**

Most MCA lesions typically exhibit clear borders and irregular edges, with some displaying expansive growth and compression of surrounding tissues. Mammography can reveal calcified components in lesions. Ultrasound often reveals an isoechoic or hypoechoic mass with well-defined borders but irregular edges. Magnetic resonance imaging (MRI) can show clear boundaries and uneven enhancement of the lesions, and the time-intensity curve (TIC) of the mass area often shows an inflow enhancement pattern.

## 1 INTRODUCTION

The primary mucinous cystadenocarcinoma (MCA) represents an exceptional and uncommon form of invasive breast cancer. Breast cancer is broadly divided into invasive breast cancer and non-invasive breast cancer.MCA was identified as a distinct subtype of mucin-secreting carcinoma in the 2002 World Health Organization (WHO) classification of Pathology and Genetics of Breast and Female Reproductive Organs. However, the classification of MCA was removed from the 2012 WHO classification, possibly due to the low incidence of MCA. The invasive breast cancer category in the 2019 WHO classification of breasts included MCA individually. Pathologically, this specimen exhibits a cystic structure when observed under the microscope. The cyst's inner lining comprises flat, tall columnar cells containing abundant intracytoplasmic and extracytoplasmic mucin arranged in layers, clusters, and papillary shapes. MCA is usually a negative expression for estrogen receptor (ER), progesterone receptor (PR), and human epidermal growth factor receptor-2 (HER-2), hence classified as triple-negative breast cancer (TNBC). However, despite sharing the triple-negative immunophenotype with traditional TNBC, MCA generally demonstrates a better prognosis. Lymph node metastasis is less common in MCA cases compared to other forms of breast cancer. Early detection of breast diseases is based on imaging examination results. However, the imaging characteristics of MCA often lack specificity, and due to the limited number of reported cases, the accuracy of early imaging diagnosis remains low. This report presents the imaging findings of a rare MCA case while analyzing and summarizing existing literature to provide valuable insights for imaging diagnosis and improve diagnostic accuracy.

## 2 CASE PRESENTATION

**Fig. (1):**
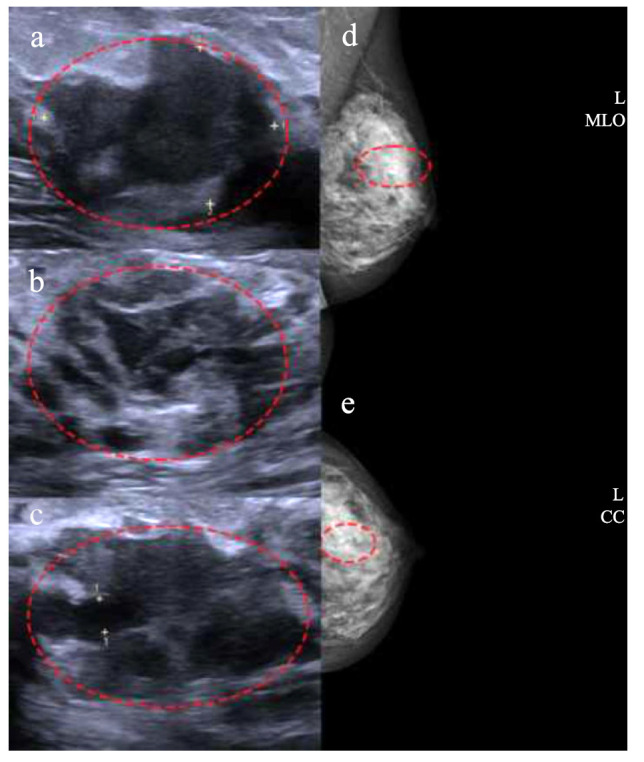
Ultrasound and X-ray images of a 40-year-old woman with primary mucinous cystadenocarcinoma (MCA) combined with invasive ductal carcinoma (IDC) in the left breast. (**a**) Ultrasonography revealed a mixed echo area in the upper outer quadrant of the left breast, measuring approximately 60 × 17 × 40mm. The lesion displayed unclear borders and an irregular shape, with an irregular liquid dark area in the center and a lack of internal sound conduction. (**b**) Multiple ductal dilations were observed surrounding the lesion. (**c**) The dilated part of the duct measured about 4 mm and appeared connected with the mixed echo area. (**d**, **e**) Mammography Mediolateral Oblique (MLO) images and craniocaudal (CC) images depicted amorphous suspicious calcifications in the middle of the upper outer quadrant of the left breast, distributed segmentally. (The area within the red circle indicates the lesion area).

**Fig. (2):**
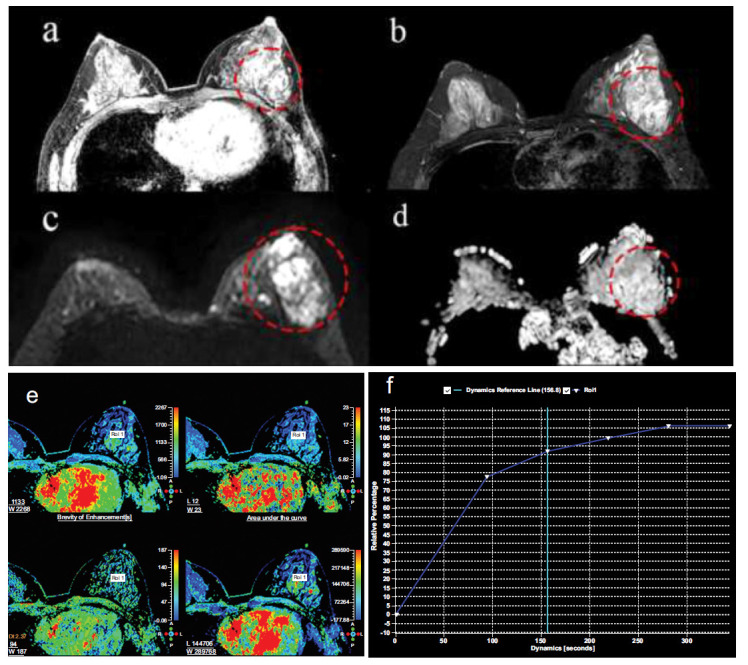
Results of the multi-parameter MRI examination of the patient's breast. (**a**, **b**) The lesion in the upper outer quadrant of the left breast showed non-mass enhancement, with a size of approximately 21 × 24mm, blurred edges, irregular shapes, lobulated edges, and burrs. (**c**) Diffusion-weighted imaging (DWI): The mass area shows a high signal. (**d**) Apparent diffusion coefficient (ADC): The mass shows a high signal, and a low signal can be observed at the edge. (**e**) Pseudo-colour parameter map of the left breast. (**f**) The mass area's time-intensity curve (TIC) shows a rapid-rising plateau type. (The area shown in the red circle is the lesion area).

The patient, a 40-year-old female with no family history of breast disease, underwent a physical examination five years ago, revealing multiple cystic nodules in the left breast. No local skin redness, swelling, depression, nipple bleeding, or discharge were observed. She was diagnosed with numerous cystic nodules of the breast and underwent regular follow-up at the local hospital. Two years ago, a breast ultrasound review indicated enlargement of the cyst in the left breast, with the largest cyst measuring 27 × 17 mm, displaying irregular shape and internal separation. Color Doppler flow imaging (CDFI) revealed no significant color blood flow in the cyst area. Due to the rapid increase in breast mass, ultrasound-guided cyst biopsy and sclerotherapy were performed. The biopsy of the left breast cyst puncture fluid showed no tumor cells, and subsequent biopsy was not conducted regularly. One year later, the patient noticed significant tumor growth, with no tenderness, normal range of motion, or discomfort such as nipple discharge. Upon follow-up examination at the hospital, ultrasound revealed a mixed echo area in the upper outer quadrant of the left breast (BI-RADS 4b), considered a malignant lesion (Fig. **1**), along with multiple cystic nodules in the right breast. Mammography showed suspicious amorphous calcifications in the middle of the upper outer quadrant of the left breast, distributed segmentally, classified as BI-RADS 5. Further dynamic contrast-enhanced breast MRI indicated non-mass enhancement (NME) in the upper outer quadrant of the left breast, diagnosed as breast cancer (BI-RADS 5), along with bilateral mastopathy, adenosis nodules, and multiple cysts in both breasts (Fig. **2**). Consequently, the patient underwent an ultrasound-guided histopathological examination, confirming invasive carcinoma in the left breast mass.

**Fig. (3):**
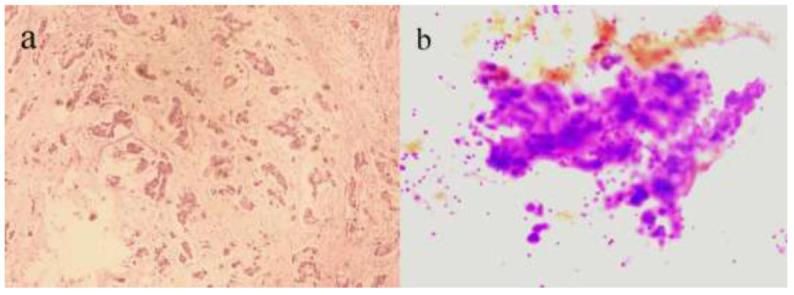
Pathological findings of the patient's left breast mass and axillary lymph node. (**a**) The postoperative pathology of left breast cancer nipple-areolar subcutaneous gland resection: invasive ductal carcinoma combined with mucinous cystadenocarcinoma. (HE, 10 ×) (**b**) The pathology results of the core needle biopsy from the left axillary lymph node: metastatic adenocarcinoma (H&E, 10 ×).

Based on the patient's relevant examination results and TNM staging standards, she was classified as T3N0M0, indicating a locally advanced tumor without lymph node or distant metastasis. Consequently, the patient underwent left breast cancer nipple-areolar subcutaneous gland resection and axillary sentinel lymph node biopsy. Intraoperatively, a yellowish-white mass in the left breast was observed, measuring approximately 12 × 11cm. The intraoperative frozen pathology report suggested a mucinous cystic tumor with severe ductal epithelial atypia, focal suspicious infiltration, and consideration of mucinous cystadenocarcinoma (Fig. **3**).

Five sentinel lymph nodes in the left breast were also removed, with no evidence of metastasis upon examination. The final pathology of the postoperative specimen confirmed invasive ductal carcinoma with some components of mucinous cystadenocarcinoma, along with vascular invasion. Immunohistochemical analysis revealed negative expression of ER and PR, HER-2 negativity, and a high Ki-67 proliferation index of 30%. Additionally, cytokeratin 5/6 (CK 5/6), globin transcription factor 3 (GATA 3), e-cadherin (E-Cad), and P120 catenin were positive. At the same time, androgen receptor (AR), P 63, smooth muscle myosin heavy chain (SMMHC), S-100 protein, SOX 10 gene, and Pax-8 gene were negative. Following surgery, the patient underwent AC-T chemotherapy for a total of eight cycles. Despite regular follow-up, an ultrasound examination revealed multiple abnormal lymph nodes in the left axilla four months post-operation. Ultrasound-guided biopsy cytology confirmed metastatic adenocarcinoma, leading to subsequent left axillary lymph node dissection and biopsy, which confirmed metastatic adenocarcinoma. The patient then received radiotherapy, resulting in stabilization of her condition.

## 3 DISCUSSION AND SUMMARY

Primary MCA of the breast is a rare malignant tumor, with a limited number of cases reported in the medical literature. The earliest documented instances date back to 1998 when the first author reported four cases of primary MCA of the breast. Since then, additional cases have been reported, with 37 cases documented as of March 2024, based on searches conducted in databases such as PubMed, Web of Science, and ProQuest. Among the reported cases, 16 cases involved primary MCA mixed with invasive ductal carcinoma (IDC) or ductal carcinoma *in situ* (DCIS) in the breast [1-11]. The average size of the masses associated with primary breast MCA is approximately 4.9cm, indicating that these tumors are typically large and often palpable by patients. Imaging modalities such as ultrasound, mammography, and MRI can aid in the early detection of these tumors. The average age of patients diagnosed with primary MCA of the breast is reported to be 61 years old, with most cases occurring in postmenopausal women [12]. However, the patient in this case study was notably younger at 39 years old, making her the youngest patient reported thus far with primary breast MCA. This highlights the importance of considering primary MCA in the differential diagnosis of breast tumors, even in younger patients, and underscores the need for further research and awareness of this rare malignancy.

According to previous reports, the ultrasound of MCA appears as isoechoic or hypoechoic masses with clear borders but irregular edges [6, 7, 11, 13, 14], and some also appear as cystic and solid masses [15-18]. Ultrasound imaging revealed a cystic-solid mass with an irregular shape, with dilated ducts visible around and inside the mass. A fluid dark area with poor sound transmission was observed within the mass, suggesting a correspondence to its microscopic structure. Microscopic examination of the breast MCA showed an unencapsulated cystic-solid mass, characterized by tall columnar tumor cells arranged in a stratified or papillary pattern, with mucin exuding from the stroma to form a mucus lake [10], the variation in internal echo performance could potentially be linked to the level of mucin exudation and accumulation. Furthermore, there are instances where focal ductal carcinoma *in situ* is observed surrounding the tumor [1-3, 10]. *In situ*, the ultrasound features of ductal carcinoma include duct dilation, intraductal hypoechoic structures, duct wall thickening, irregular duct caliber, and echogenic foci in the duct lumen. The dilated ducts surrounding the lesion are likely associated with ductal carcinoma. Differential diagnosis between intraductal papillary carcinoma (IPC) and MCA can be challenging. IPC may present as a cystic-solid mass on ultrasound, sometimes causing duct blockage and showing hypoechoic mass and local duct dilation on ultrasound. Superficial mucinous breast cancer may appear as a mixed echogenic cystic-solid mass with a slightly lobulated appearance.

However, mucinous carcinomas typically exhibit expansive growth with colloid-like material separated by fibers, distinguishing them from MCA. Mammographic images of MCA normally depict well-defined lobulated masses [1, 4, 11, 19, 20] with medium to high density and irregular calcifications [6, 13]. In this case, no apparent space-occupying lesions were observed on mammography, with suspicious amorphous calcifications detected only in the upper outer quadrant of the left breast in a segmental distribution. MRI findings of MCA commonly depict a low signal on the T1 weighted image (T1WI), an uneven high signal on the T2 weighted image (T2WI), uneven enhancement of the solid part, and continuous enhancement in the dynamic enhanced imaging time-signal intensity curve (TIC) [11, 15, 21, 22]. In this case, the contrast-enhanced MRI depicted non-mass enhancement characterized by unclear boundaries, irregular shape, lobulated and spiculated edges, and a fast-rising plateau type in the time-intensity curve (TIC). The lesion exhibited blurred edges, lacked a clear boundary in enhancement, and manifested a type II dynamic enhancement pattern. It is considered that this lesion might be associated with invasive breast cancer.

In summary, the imaging characteristics of MCA are diverse (Table **1**). Most MCA lesions typically present with clear borders and irregular edges, while some may demonstrate expansive growth leading to compression of surrounding tissues. Various imaging modalities play a crucial role in the diagnosis of MCA. Mammography delineates the shape, number, and distribution of calcifications, providing insights into malignant tendencies. Ultrasound often depicts an isoechoic or hypoechoic mass with well-defined borders but irregular edges. MRI can delineate lesion boundaries and demonstrate inflow-type enhancement patterns on the TIC curve, typically suggestive of a benign tumor.

Mammography is particularly sensitive to calcifications within lesions, with different types of calcifications often indicating varying benign and malignant tendencies. This sensitivity is especially significant when the lesion is associated with other malignant pathological components, as mammography can provide early indications of malignancy. Moreover, ultrasound offers distinct advantages in assessing the shape and boundaries of lesions; it can also clearly display the formation of mucus lakes within tumors. In addition, Magnetic resonance imaging provides additional information for evaluating the tissue composition of lesions, and the time-intensity curve of the lesion area can further indicate the benign or malignant nature of the lesion to some extent. Consequently, adopting a comprehensive approach integrating multiple imaging modalities is imperative to achieve early characterization and prevent the misdiagnosis of benign conditions. Moreover, given the limitations of relying solely on imaging findings, combining ultrasound-guided targeted tissue puncture with pathological examination becomes essential. In the pathological assessment of primary MCA of the breast, accurate differentiation from other carcinoma types like breast mucinous carcinoma and encapsulated papillary carcinoma is critical. Additionally, distinguishing mucinous cystadeno- carcinoma from alternative sources can pose challenges, necessitating immunohistochemical staining such as cytokeratin 20 (CK20), caudal type homeobox 2 gene (CDX2), and trichorhinophalangeal syndrome type 1 (TRPS1) to exclude alternative origins [23]. Surgical intervention remains the primary approach for managing breast MCA, complemented by chemotherapy and local radiotherapy for recurrent or metastatic cases.

Additionally, targeted therapy has emerged as a cornerstone treatment for mid-to-late-stage breast MCA. Research has identified phosphatidylinositol-4,5-bisphosphate 3-kinase catalytic subunit alpha (PIK3CA) as a pivotal therapeutic target, with drugs tailored to this target progressing to the clinical research stage [23].

## CONCLUSION

Indeed, primary mucinous cystadenocarcinoma of the breast is rare, and imaging reports are scarce. The objective of this study is to comprehensively analyze and summarize the imaging characteristics of these tumors using ultrasound and magnetic resonance images. By doing so, we aim to improve the precision of imaging-based diagnoses for this specific type of tumor and provide valuable insights and methodologies for the early detection of this disease in future clinical practice.

## Figures and Tables

**Table 1:** Imaging performance summary for primary mucinous cystadenocarcinoma.

Age(years)	TNM	Pathological Results	Mammographic Findings	Magnetic Resonance Image Finding	Ultrasonographic Findings	Reference
65	T2N0M0	MCA, IDC&DCIS	Well-defined, medium to high density, multilobulated mass	N.A.	Circumscribed, isoechoic to hypoechoic lesion	[1]
55	T2N0M0	MCA, IDC&DCIS	Round, isodense, circumscribed mass	N.A.	Circumscribed lesion with complex hypoechoic to isoechoic contents	[2]
73	T2N0M0	MCA, DCIS	NA	NA	Lobulated, heterogenous internal echogenicity	[3]
65	T2N0M0	MCA, DCIS	Well-defined, medium to high density, lobulated mass	N.A.	Circumscribed, isoechoic to hypoechoic lesion	[4]
59	T1N0M0	MCA, IDC&DCIS	Focal asymmetry	N.A.	Hypoechoic mass with a speculated margin	[5]
41	T3N1M0	MCA, DCIS	Patchy irregular calcifications within the masses	N.A.	Irregularly shape lesion	[6]
68	T3N0M0	MCA, DCIS	Nodular enhancing lesion	NA	Well-circumscribed, solid, and cystic breast mass with some irregular margins and mixed echogenicity	[7]
69	T1N0M0	MCA, DCIS	Hyperdense mass	N.A.	lobulated hypoechoic mass	[8]
45	T2N0M0	MCA, DCIS	NA	NA	Slightly heterogenous hypoechoic mass with irregular micro-lobular margins	[9]
66	T2N0M0	MCA, DCIS	High-density shadow, the edge was rough	N.A.	Hypoechoic mass with multiple hypoechoic nodules around it, and the border was unclear.	[10]
61	T2N1M0	MCA, IDC&DCIS	Irregular lobulated mass	Low signal intensity on T1-weighted imaging and high signal intensity on T2-weighted imaging, irregular rim enhancement and a spiculated boundary.	Cystic-solid lump with a clear boundary and irregular contour	[11]
61	T2N2M0	MCA	NA	NA	A well-defined oval-shaped, high-density mass with a cystic component in the retro areolar region is associated with architectural distortion.	[12]
79	T3N0M0	MCA	The patchy irregular calcification within the masses	N.A.	Irregularly shaped	[13]
72	T1N0M0	MCA	Oval mass with obscured margins	N.A.	Heterogeneous irregular mass with circumscribed margins	[14]
59	T1N0M0	MCA	Complex cystic and solid masses	A cystic portion of the masses showed a circumscribed margin and thin rim enhancement, while the intracystic solid portion exhibited nodular enhancement and a large irregular solid mass with heterogeneous enhancement. The dynamic enhancement images revealed enhancement kinetics; in T2-weighted imaging, the signal intensity was intermediate.	Cystic and solid masses	[15]
74	T3N0M0	MCA	NA	NA	Multilocular and lobulated tumour with a focal solid growth	[16]
62	T2N0M0	MCA	NA	NA	Well-circumscribed and lobulated cystic-solid mass	[17]
56	T1N0M0	MCA	Abnormity high-density shadow	Irregular masses and calcification.	Multiple ectatic ducts accompany cystic and solid masses.	[18]
52	T3N0M0	MCA, ADH	Well-defined multilobular mass	N.A.	NA	[19]
52	T3N0M0	MCA	Well-defined, medium to high density, multilobulated mass	N.A.	NA	[20]
50	T3N0M0	MCA	Well-defined	The lesion demonstrated inhomogeneous hyperintensity on the MR T2-weighted sagittal image, hypointensity on the T1-weighted axial image, and heterogeneous persistent enhancement kinetics on the dynamic contrast-enhanced axial image.	NA	[21]
65	T4N1M1	MCA	NA	Heterogenous enhancing soft tissue density lesion	NA	[22]

## Data Availability

All data generated or analyzed during this study are included in this article. Further inquiries can be directed to the corresponding author [Y.Z] upon reasonable request.

## LIST OF ABBREVIATIONS

ADC: Apparent diffusion coefficient
AR: Androgen receptor
CC: Craniocaudal
CK20: Cytokeratin 20
CDFI: Color doppler flow imaging
CDX2: Caudal type homeobox 2 gene
CK5/6: Cytokeratin 5/6
DCIS: Ductal carcinoma *in situ*
DWI: Diffusion weighted imaging
E-cad: E-cadherin
ER: Estrogen receptor
GATA3: Globin transcription factor 3
HER-2: Human epidermal growth factor receptor-2
IDC: Invasive ductal carcinoma
IPC: Intraductal papillary carcinoma
MCA: Mucinous cystadenocarcinoma
MLO: Mediolateral oblique
MRI: Magnetic resonance imaging
NME: Non-mass enhancement
PIK3CA: Phosphatidylinositol-4,5-bisphosphate 3-kinase catalytic subunit alpha
PR: Progesterone receptor
SMMHC: Smooth muscle myosin heavy chain
T1WI: T1 weighted image
T2WI: T2 weighted image
TIC: Time-signal intensity curve
TNBC: Triple-negative breast cancer
TRPS1: Trichorhinophalangeal syndrome type 1

## STANDARDS OF REPORTING

CARE guidelines were followed.
